# Carbohydrate counting as a strategy to optimize glycemic control in type 1 diabetes mellitus

**DOI:** 10.20945/2359-3997000000596

**Published:** 2023-02-07

**Authors:** Analaura Centenaro, Cigléa do Nascimento, Mileni Vanti Beretta, Ticiana da Costa Rodrigues

**Affiliations:** 1 Universidade Federal do Rio Grande do Sul Faculdade de Medicina Programa de Pós-graduação em Ciências Médicas: Endocrinologia Porto Alegre RS Brasil Programa de Pós-graduação em Ciências Médicas: Endocrinologia, Faculdade de Medicina, Universidade Federal do Rio Grande do Sul (UFRGS), Porto Alegre, RS, Brasil; 2 Hospital de Clínicas de Porto Alegre Divisão de Endocrinologi Porto Alegre RS Brasil Divisão de Endocrinologia, Hospital de Clínicas de Porto Alegre (HCPA), Porto Alegre, RS, Brasil; 3 Universidade Federal do Rio Grande do Sul Faculdade de Medicina Departamento de Clínica Médica Brasil Programa de Pós-graduação em Ciências Médicas: Endocrinologia, Departamento de Clínica Médica, Faculdade de Medicina, Universidade Federal do Rio Grande do Sul (UFRGS); Divisão de Endocrinologia, Hospital de Clínicas de Porto Alegre (HCPA), Porto Alegre, RS, Brasil; Hospital de Clínicas de Porto Alegre Divisão de Endocrinologia Porto Alegre RS Brasil

**Keywords:** Carbohydrate counting, type 1 diabetes mellitus, glycemic control, glycated hemoglobin, body weight

## Abstract

**Objective:**

The objective of this study was to verify the impact of carbohydrate counting (CC) on glycemic control and body weight variation (primary and secondary outcomes, respectively) between consultations in patients with diabetes mellitus (T1D) followed at a tertiary hospital in southern Brazil in a public health system environment. We also sought to investigate CC adherence.

**Materials and methods:**

This retrospective cohort study included 232 patients with T1D who underwent nutritional monitoring at a referral hospital for diabetes care between 2014 and 2018. To assess primary and secondary outcomes, data from 229 patients, 49 of whom underwent CC during this period and 180 individuals who used fixed doses of insulin, were analyzed. The impact of CC on glycemic control was assessed with the mean glycated hemoglobin (HbA1c) level at all consultations during the follow-up period.

**Results:**

In the model adjusted for the most confounders (except pregnancy), the mean HbA1c was better in the CC group (8.66 ± 0.4% vs. 9.36 ± 0.39%; p = 0.016), and body weight variation was lower (0.13 ± 0.28 kg vs. 0.53 ± 0.24 kg; p = 0.024). Adherence to CC was reported in 69.2% of consultations.

**Conclusion:**

CC optimized the glycemic control of individuals with T1D, resulting in less weight variation than in the fixed insulin dose group, which indicates that CC is an important care strategy for these patients.

## INTRODUCTION

Treating type 1 diabetes mellitus (T1D) is a challenge for patients, their families, and multidisciplinary care teams due to the disease's characteristics, the use of insulin and the constant monitoring of blood glucose levels. Hyperglycemia exposes individuals to the risk of developing chronic complications (1-3), which are associated with considerable rates of morbidity, mortality and high health costs (4,5). However, long-term observational follow-up studies and clinical trials have demonstrated that adequate glycemic control reduces the incidence of microvascular and macrovascular disease (6-9). Therefore, different treatment approaches that optimize glycemic control should be explored, including carbohydrate counting (CC) (10). This technique focuses on the amount of carbohydrates (CHO) consumed (11), since this nutrient is the major determinant of the postprandial glycemic response (12).

Divergent results have been found in previous studies on CC as a strategy for optimizing glycemic control in individuals with T1D. While some studies have reported that CC has no effect on glycated hemoglobin (HbA1c) compared to a control group (13-16), others have found that the intervention improved control (17-20). The greatest difference was found in the DAFNE study: an approximately 1% difference in HbA1c reduction (18). However, most of these studies did not have a long follow-up period [duration between 3.5 and 30 months; only two > 1 year (17,20). Some short-term Brazilian studies have also found that CC optimizes glycemic control, although none were conducted in the southern region of the country (21,22).

Because CC provides dietary flexibility by allowing bolus dose adjustments according to CHO consumption, which could additionally result in higher doses of insulin (18,23,24), investigating the effect of this technique on body weight is also important, since obesity is associated with a less favorable cardiometabolic profile (25). However, most studies have not associated CC with weight (16-18) or body mass index (BMI) (17,20,26), although some data indicate that CC leads to a reduced BMI (23).

In view of such evidence, the aim of the present study was to evaluate the effects of CC on glycemic control and variations in body weight between consultations in T1D patients treated at a tertiary hospital in southern Brazil in a real-life public health care model. We also sought to assess CC adherence.

## MATERIALS AND METHODS

This retrospective cohort study included all patients (children, adolescents, adults, older adults, and pregnant women) diagnosed with T1D who had consultations with the dietitian at the outpatient endocrinology clinic of a university hospital, referral in diabetes care, between January 2014 and December 2018. A total of 326 potentially eligible patients were identified through the hospital's electronic records of consultations during the period. We excluded a total of 94 patients who, during the study period, had only one nutritional consultation (n = 80), received less than 3 months of nutritional follow-up (n = 13), or underwent CC for less than 3 months (n = 1). Thus, the final sample consisted of 232 patients. To assess glycemic control and body weight change between consultations (primary and secondary outcomes, respectively), the patients were divided into two groups: a group that only underwent conventional nutritional monitoring but not CC (n = 180) and used fixed doses of insulin and a group that performed CC between 2014 and 2018 (n = 52). Patients in the second group could have begun CC before or during the study period or interrupted it between 2014 and 2018. Thus, since some CC group patients were using fixed doses of insulin at the time of one or more consultations, only data from the period in which the patients were actually performing CC were included in the primary and secondary outcome analysis, and only these consultations were considered as the follow-up time in the analysis. As a result, 3 additional individuals were excluded from this assessment, since only one nutritional consultation could be analyzed; hence, the CC group included a total of 49 individuals. However, to determine adherence to CC, data for all 52 patients who underwent the technique were included, and only consultations between 2014 and 2018 in which CC was actually performed were considered.

Patients who underwent CC were trained by the outpatient dietitian (27). The dose of insulin bolus to be applied at the meal was calculated using the following formula (28):


Total bolus=meal bolus (MB)+correction bolus (CB),

where

MB = grams of carbohydrate in the meal/insulin-to-carbohydrate ratio (ICR)

CB = (preprandial glucose - glycemic target)/insulin sensitivity factor (ISF)

The ICR indicates the grams of carbohydrates metabolized by one unit of insulin (UI), while the ISF reports the blood glucose reduction (mg/dL) for each administered UI (29,30). Initially, the ICR was estimated as 500 divided by the total daily insulin dose (TDID), while the ISF was calculated as 1,500 or 1,800 (for short-acting insulin or rapid-acting insulin analogs, respectively) divided by the TDID, with adjustments in the ICR and the ISF when necessary being made at follow-up consultations based on patient-recorded glycemic controls and insulin doses (28,30-33). Changes in basal insulin doses were made by an endocrinologist at the diabetes clinic.

Patients who underwent nutritional monitoring but not CC received individual nutritional guidance at each consultation and administered fixed doses of bolus insulin adjusted only to the preprandial blood glucose value; these doses were also prescribed by an endocrinologist.

The data were extracted entirely from the electronic records of each patient. The following sociodemographic data were collected: sex, ethnicity, and maximum education level reported during the follow-up period (classified as ignored, none, elementary/high school/higher education or graduate school – complete or incomplete). The following clinical data were also collected: date of T1D diagnosis and date of initial nutritional monitoring and CC.

The following repeated measures variables were collected at each nutritional consultation between January 2014 and December 2018: CC (yes vs. no), pregnancy (yes vs. no – adult and adolescent women), medications used, types of insulin administered (basal: intermediate-acting or long-acting analogs; bolus: short-acting or rapid-acting analogs), daily dose per kg of body weight and self-monitoring of capillary blood glucose (SMBG) as recommended (yes vs. no). For the latter, three daily measurements were requested (before breakfast, before lunch and before dinner) and two hours postprandial seven days before the consultation (ideally at all meals, but if the patient did not have enough strips, patients took measurements each day in alternate meals – one day after breakfast, another after lunch, and in the other after dinner). The following laboratory tests performed for routine consultations were verified: plasma levels of fasting glucose, HbA1c, total cholesterol (TC), high-density lipoprotein cholesterol (cHDL), and triglycerides (TG), as well as albuminuria from urine samples. The low-density lipoprotein cholesterol (cLDL) concentration was calculated using the Friedewald formula: cLDL = TC – (cHDL + TG/5) when TG levels < 400 mg/dL (34). Kidney function was determined by calculating the glomerular filtration rate (GFR) using the CKD-EPI formula for adults and Schwartz's method for children and adolescents (35). For anthropometric evaluation, weight and height were collected to calculate BMI using the formula weight/height², and nutritional status was evaluated using the cutoff points recommended for adults (36), elderly individuals (37) and pregnant women (38). For children and adolescents, the WHO Anthro and WHO AnthroPlus software was used to calculate BMI-for-age z-scores (39,40). Patients were divided into two categories: underweight/eutrophic vs. excess weight (overweight or obesity). The variation in body weight in relation to that of the previous nutritional consultation was also calculated. To assess the patients’ physical activity level, the total time of activity (minutes) per week was determined, and the individuals were then classified as either sufficiently active (≥60 minutes/day for children and adolescents and ≥ 150 min/week for adults) or insufficiently active (10).

At each nutritional consultation, patients in the CC group were asked about performing the technique according to the instructions received. Until deemed necessary (usually until assimilation of the method), the dietitian requested records of the food and quantities ingested and the calculation of corresponding CHO grams, MB and CB. Adherence to CC was assessed by the report in the dietitian's records, and patients were classified as adherent (when the dietitian reported correct CC performance according to her prescription) vs. not/partially adherent (if the professional reported not performing/partially performing the provided orientation).

Based on the information collected, age, diabetes mellitus (DM) duration, nutritional follow-up time, and time of CC were calculated for each nutritional consultation. The total number of nutritional consultations and absences between 2014 and 2018 was also calculated.

Data were also collected on comorbidities and chronic complications of DM (developed before or during the follow-up period). Complications included a medical diagnosis of retinopathy, neuropathy, diabetic kidney disease (DKD) or cardiovascular events (death from cardiovascular disease (CVD), acute myocardial infarction, stroke, and peripheral or coronary artery disease requiring revascularization or angioplasty). DKD was considered confirmed when the patient had at least two values indicating albuminuria ≥14 mg/L or a GFR < 60 mL/min/1.73 m² at least 6 months before the diagnosis of DKD (41). Comorbidities included hypertension, psychiatric diseases (depression, bulimia, panic syndrome, anxiety disorder, bipolarity, attention deficit, and hyperactivity), functional thyroid diseases (hypothyroidism or hyperthyroidism), other autoimmune diseases in addition to T1D (celiac disease, Hashimoto's thyroiditis, rheumatoid arthritis, Graves’ disease, Sjogren's syndrome and vitiligo), and eye diseases other than diabetic retinopathy (amaurosis, cataracts and glaucoma).

Due to the complexity of estimating the TDID for each patient, data imputations were made in some situations of missing or confusing values, i.e., the mean of the previous and subsequent consultation or repeating the value immediately before or after when referring to the last and first consultations, respectively. For the other variables, missing data were considered missing. The primary outcome was the effect of CC on glycemic control based on HbA1c values. As a secondary outcome, the impact of CC on body weight variation between appointments was considered. We also calculated the proportion of CC consultations in which patients were considered adherent.

This study was approved by the Research Ethics Committee of the Hospital Graduate Studies Group (protocol 2019-0079) and registered with CAAE number: 07931418.0.0000.5327. All researchers involved in the study signed the Data Use Agreement.

### Statistical analysis

Variables were analyzed as either a single measure (a single value during the follow-up period or referring to baseline, i.e., the first consultation evaluated between 2014 and 2018) or as repeated measures over the follow-up period (measured at each nutritional consultation).

For the primary and secondary outcomes, single-measure variables are presented as the mean ± standard deviation (SD), median (interquartile range P25-P75) or number of cases (%). The distribution of continuous variables was evaluated using the Shapiro-Wilk test. The *t* test, Mann-Whitney test, and chi-square test were used to compare parametric, nonparametric, and categorical variables, respectively, between the CC group and the group that used fixed doses of insulin. A linear mixed model for repeated measures, a generalized linear mixed model for repeated measures and a generalized linear mixed model for dichotomous response were used to compare parametric, nonparametric and categorical variables, respectively, measured at each nutritional consultation. In addition to the main effect of the variable (p value), its interaction with time (p for interaction) was also analyzed. Continuous variables are presented as the means ± standard errors (SE) and 95% confidence interval (95% CI), and dichotomous variables are presented as the estimated proportion (%) ± SE and 95% CI. Variables whose effect was not constant during the follow-up period (p for interaction < 0.05) only have this effect cited in the text, since the values are not the same in the different periods and the follow-up time was treated as a continuous variable in this analysis (number of weeks elapsed between each nutritional consultation during the follow-up period and the baseline consultation); therefore, it varies among patients, with considerable extension and variability of values.

For the CC adherence analysis, we calculated the frequency (%) of consultations (among those in which CC was performed) in which patients were classified as compliant.

Although education was included in the between-group analyses (CC vs. fixed insulin doses) in all categories (ignored, none, elementary school/high school/higher education or graduate school – complete or incomplete), it is presented as ignored/≤ completed elementary school, high school, or ≥ incomplete higher education.

All repeated measures analyses were adjusted for the duration of the patients’ nutritional follow-up at baseline, and for the time. Multivariate models were developed based on univariate results and clinical judgment. As the number of pregnant women differed between groups (CC vs. fixed doses of insulin), this variable was included in the multivariate analysis; however, the number of individuals in this model was significantly reduced (only adult and adolescent women); therefore, an analysis with the total sample, excluding consultations during pregnancy, was also carried out.

P values < 0.05 were considered statistically significant. The analyses were performed in SPSS, version 22.0 (IBM Corp, Armonk, NY).

## RESULTS

The median follow-up time was 105 (43-198) weeks. Table 1 compares the single-measure variables between the groups (CC vs. not CC), including sociodemographic and clinical characteristics, the number of pregnant women, and the number of consultations. Regarding ethnicity, the percentage of whites was higher in the CC group than in the group using fixed doses of insulin [49 (100%) vs. 161 (89.4%); p = 0.045]. The CC group also had a higher education level (i.e., more patients with at least incomplete higher education and fewer with an ignored education degree or with no more than primary education p = 0.001). In addition, the CC group had more pregnant women [5 (10.2%) vs. 1 (0.55%); p = 0.003] and longer nutritional follow-up at baseline [97 (5.5-129.5) vs. 43.5 (0-126.75) months]. The total number of nutritional consultations carried out between 2014 and 2018 was also higher in the CC group [10 (6-14) vs. 5 (3-9); p = 0.000]. The other variables did not significantly differ between groups.

**Table 1:** Sociodemographic and clinical characteristics, number of pregnant women and number of consultations in the study population (single-measure variables)

Variable		CC (n = 49)	Not CC (n = 180)	p value
Age, years§		32.91 ± 11.31	30.7 ± 16.26	0.275
Nutritional follow-up time, months§		97 (5.5-129.5)	43.5 (0-126.75)	0.045
Diabetes duration, months§		195 (112-265)	147 (33.5-266.55)	0.1
BMI (kg/m²)§		24.4 ± 4.26	23.6 ± 4.7	0.284
Age at DM diagnosis, years		16 (10.5-24.5)	14 (8.25-25)	0.588
Males (n, %)		20 (40.8)	84 (46.7)	0.570
Ethnicity (n, % white)		49 (100)	161 (89.4)	0.045
Maximum education (n, %)				
	Ignored/≤ complete elementary	15 (30.7)	86 (47.8)	
	High school	15 (30.6)	68 (37.7)	
	≥ Incomplete higher education	19 (38.7)	26 (14.5)	0.001
Pregnant women (n, %)+		5 (10.2)	1 (0.55)	0.003
Number of consultations#		10 (6 - 14)	5 (3 - 9)	0.000
DKD (n, %)*		1 (2)	14 (7.8)	0.202
GFR < 60 (mL/min/1.73 m²) (n, %)*		1 (2)	15 (8.3)	0.204
Positive albuminuria (n, %)*		38 (77.6)	127 (70.6)	0.431
Neuropathy (n, %)*		2 (4.1)	15 (8.3)	0.538
Retinopathy (n, %)*		23 (46.9)	60 (33.3)	0.112
Cardiovascular events (n, %)*		1 (2)	1 (0.6)	0.383
Ocular diseases (n, %)*		1 (2)	15 (8.3)	0.204
Autoimmune diseases (n, %)*		10 (20.4)	34 (18.9)	0.972
Psychiatric disorders (n, %)*		10 (20.4)	41 (22.8)	0.873
Thyroid diseases (n, %)*		13 (26.5)	38 (21.1)	0.539
Hypertension (n, %)*		8 (16.3)	42 (23.3)	0.391

CC: carbohydrate counting; BMI: body mass index; DKD: diabetic kidney disease; DM: diabetes mellitus; GFR: glomerular filtration rate; SD: standard deviation

Data are presented as mean ± SD, median (interquartile range P25-P75) or number and percentage of cases (n, %).

§ Referring to baseline (first consultation included during the period 2014 and 2018).

+ Number along follow-up consultations.

# All consultations between 2014 and 2018 were considered in the CC group, including those in which patients used fixed doses of insulin.

* Occurring prior to or during the follow-up period.

Table 2 compares repeated measurements of clinical, laboratory and anthropometric variables. SMBG was performed more frequently in the CC group (92.2 ± 2.4 vs. 84.4 ±1.8; p value = 0.005), and the use of rapid-acting insulin analogs was also more common in this group (100 ± 0 vs. 77.9 ± 3.1; p value = 0.000). BMI and the proportion of patients classified as sufficiently vs. insufficiently active also significantly differed between the two groups, although these values were not constant throughout follow-up (p for interaction = 0.008 and 0.005, respectively).

**Table 2:** Clinical, laboratory and anthropometric characteristics of the study population (repeated-measures variables)

Variable		CC (n = 49)	Not CC (n = 180)	p value§	p for interaction¶
Fasting glucose (mg/dL)*		203.42 ± 11.23 (182.53-226.7)	207.87 ± 5.93 (196.55-219.84)	0.918	0.741
Total cholesterol (mg/dL)*		178.81 ± 7.17 (165.24-193.5)	186.42 ± 3.96 (178.78-194.39)	0.522	0.761
cHDL (mg/dL)*		58.11 ± 2.87 (52.72-64.05)	58.98 ± 1.57 (55.97-62.15)	0.61	0.606
cLDL (mg/dL)*		102.72 ± 5.57 (92.31-114.31)	106.39 ± 3.11 (100.42-112.71)	0.458	0.684
Triglycerides (mg/dL)*		88.44 ± 8.9 (72.53-107.83)	97.31 ± 5.3 (87.41-108.33)	0.91	0.106
GFR (mL/min/1.73m²)*		102.4 ± 4.57 (93.79-111.81)	99.03 ± 2.25 (94.71-103.56)	0.654	0.615
SMBG**					
	Yes	92.2 ± 2.4 (86-95.7)	84.4 ± 1.8 (80.6-87.6)	0.005	0.127
Insulin dose* (UI/kg)		0.66 ± 0.03 (0.6-0.73)	0.71 ± 0.02 (0.67-0.75)	0.199	0.607
Basal insulin**					
	Long-acting analogs	60.7 ± 6.8 (46.9-73)	42.4 ± 3.6 (35.6-49.5)	0.055	0.129
Bolus insulin**					
	Rapid-acting analogs	100 ± 0 (82.1-100)	77.9 ± 3.1 (71.3-83.4)	0.000	0.163
Nutritional status**					
	Excess body weight	40 ± 6.5 (28.4-53.5)	43 ± 3.5 (36.4-50)	0.813	0.653

CC: carbohydrate counting; cHDL: high-density lipoprotein cholesterol; cLDL: low-density lipoprotein cholesterol; GFR: glomerular filtration rate; SE: standard error; SMBG: self-monitoring of capillary blood glucose; UI: unit of insulin

* Continuous variables presented as mean ± SE and 95% CI.

** Dichotomous variables presented as estimated proportion (%) ± SE and 95% CI.

§ p value of the effect of the variable.

¶ p of interaction between the effect of the variable and time.

All analyses were adjusted for the nutritional follow-up time that the patients already had at baseline, as well as for the time elapsed between each consultation during the study period (2014 and 2018) and baseline.

**Figure 1:**
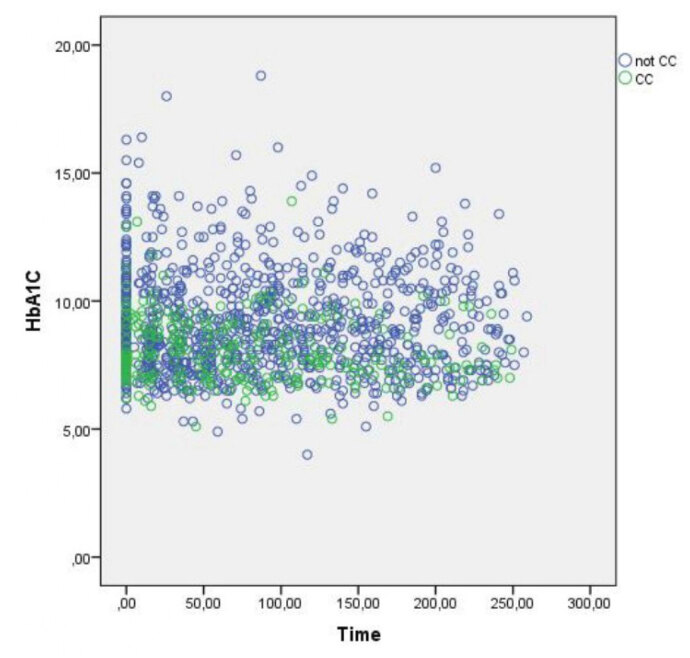
HbA1c in both groups during the follow-up period. Values collected at each nutritional consultation analyzed between 2014 and 2018. Time: weeks from baseline; HbAlc: Glycated Hemoglobin.

HbA1c values collected in both groups at each nutritional consultation analyzed during follow-up are shown in Figure 1. Table 3 shows the association between CC and glycemic control during the follow-up period. After adjusting for most confounding variables (Model 1), the mean HbA1c was significantly lower in the CC group than in the fixed doses of insulin group (8.66 ± 0.4% vs. 9.36 ± 0.39%, p value = 0.016), and this difference was constant over time (p for interaction = 0.841). When performing an additional adjustment for pregnancy (Model 2), a lower mean HbA1c was observed in the CC group (8.26 ± 0.58% vs. 8.82 ± 0.55%), but this difference was not significant (p value = 0.107 and p for interaction = 0.999).

**Table 3:** Association between CC and glycemic control

HbA1c (%)*	CC (n = 49)	Not CC (n = 180)	p value§	p for interaction¶
Model 0	8.2 ± 0.21 (7.8 to 8.63)	9.13 ± 0.12 (8.89 to 9.38)	0.000	0.735
Model 1	8.66 ± 0.4 (7.9 to 9.5)	9.36 ± 0.39 (8.62 to 10.16)	0.016	0.841
Model 2	8.26 ± 0.58 (7.19 to 9.49)	8.82 ± 0.55 (7.8 to 9.98)	0.107	0.999

CC: carbohydrate counting; HbA1c: glycated hemoglobin; SE: standard error.

* HbA1c measured at each nutritional consultation during follow-up (2014 and 2018); presented as mean ± SE and 95% CI.

Model 0: adjustment for the nutritional follow-up time that the patients already had at baseline and for the time elapsed between each consultation during the study period (2014 and 2018) and baseline.

Model 1: model 0 + adjustment for ethnicity (white, black, mixed race), education (ignored, none, elementary/high school/ higher education or graduate school – complete or incomplete), total number of consultations between 2014 and 2018, BMI, SMBG (dichotomous), bolus insulin (short-acting or rapid-acting analogs) and physical activity (sufficiently or insufficiently active).

Model 2: model 1 + adjustment for pregnancy (dichotomous), only consultations with adult and adolescent women were included in this model.

§ p value of the effect of the variable.

¶ p of interaction between the effect of the variable and time.

The mean weight variation between nutritional consultations (Table 4) was positive in both groups but lower in those who performed CC (Model 3) (0.13 ± 0.28 kg vs. 0.53 ± 0.24 kg, p value = 0.024), and this difference was also constant throughout the follow-up period (p for interaction = 0.226). In an additional adjustment for pregnancy using only data from adult and adolescent women, the difference, although still significant, was not constant throughout the follow-up period (p for interaction = 0.035).

**Table 4:** Comparison of weight variation between appointments – CC vs. fixed insulin doses

Weight variation (kg)*	CC (n = 49)	Not CC (n = 180)	p value§	p for interaction¶
Model 0	0.23 ± 0.13 (-0.033 to 0.492)	0.43 ± 0.07 (0.28 to 0.58)	0.045	0.14
Model 1	0.27 ± 0.29 (-0.3 to 0.85)	0.52 ± 0.25 (0.02 to 1.02)	0.027	0.082
Model 2	0.2 ± 0.29 (-0.378 to 0.79)	0.516 ± 0.25 (0.004 to 1.02)	0.023	0.11
Model 3	0.13 ± 0.28 (-0.42 to 0.69)	0.53 ± 0.24 (0.04 to 1.02)	0.024	0.226

CC: carbohydrate counting; SE: standard error.

* Variable measured at each nutritional consultation between 2014 and 2018 and presented as mean ± SE and 95% CI.

Model 0: adjustment for the nutritional follow-up time that the patients already had at baseline and for the time elapsed between each consultation during the study period (2014 and 2018) and baseline.

Model 1: model 0 + adjustment for ethnicity (white, black, mixed race), education (ignored, none, elementary/high school/ higher education or graduate school – complete or incomplete), number of consultations between 2014 and 2018, BMI, SMBG (dichotomous) and insulin bolus (short-acting or rapid-acting analogs).

Model 2: model 1 + adjustment for physical activity (sufficiently or insufficiently active).

Model 3: model 2 + adjustment for age and sex.

§ p value of the effect of the variable.

¶ p of interaction between the effect of the variable and time.

Performing the same analyses, but excluding consultations during pregnancy, one patient was excluded from the CC group, and the significant difference for ethnicity was not maintained between groups [whites: 48 (100%) in CC vs. 161 (89.4%) in not CC; p = 0.051]. All other results were similar to those conducted with the entire sample (data not shown).

**Figure 2:**
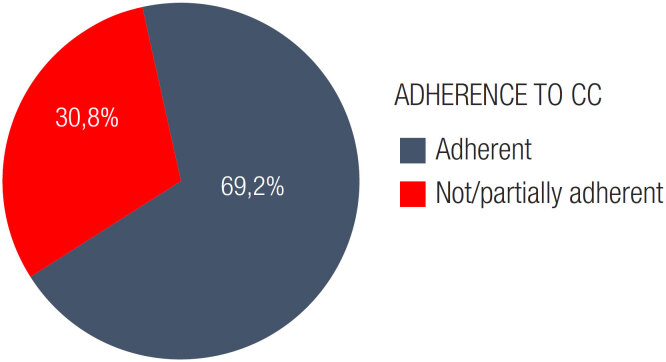
Adherence to CC. Frequency (%) of consultations (among those in which CC was performed) in which patients were classified as compliant or not/partially compliant. CC, carbohydrate counting.

Adherence to CC was reported in 69,2% of the CC consultations (Figure 2).

## DISCUSSION

In this real-life study in a public health system environment, T1D patients in the CC group had better glycemic control and less variation in body weight than the standard nutritional monitoring group, showing a difference in HbA1c with potential clinical impact (≈-0.7%).

According to previous studies, the effects of the CC method are somewhat divergent in patients with T1D (13-23,26). Of the randomized controlled trials (RCT) that compared CC to a control group, several (17-20) found that the intervention optimized glycemic control, while others did not (13-16).

Additionally, studies from 2020 and 2021 found that CC was only effective in the short term. In the 2020 study, CC resulted in a lower mean HbA1c during 1 year of follow-up, although when the analysis was performed separately at 3, 6, 9 and 12 months, the benefit was maintained only in the first evaluation (26). The 2021 study found that CC had a positive effect on HbA1c after 3 months of treatment, but not after 12 months (42). In the Brazilian population, a 4-month clinical trial of 28 adolescents from Goiás found that HbA1c was lower in the CC group than in the control group (22). A cross-sectional study of children and adolescents in Rio de Janeiro in which 80% of the sample performed CC found that the technique was associated with lower HbA1c values (21). According to our results, CC optimized HbA1c, corroborating some of these data (17-22,26) with a longer follow-up time (13-16,18-20,26,42) and superior sample size (13-18,20,26,42) to most other studies.

Adjusting for pregnancy reduced the number of consultations in the analysis, and although the difference in HbA1c was maintained, it did not remain statistically significant. However, the difference was clinically relevant, so CC may have had a significant benefit if a larger sample size had been included. Analyses that excluded consultations during pregnancy did not significantly change the results obtained with the entire sample.

A meta-analysis that included the abovementioned RCTs (except for those from 2020 and 2021) investigated the effects of CC on HbA1c in T1D. Although the quality of evidence was moderate, the intervention appeared to be associated with lower HbA1c values. Nevertheless, the magnitude of the effect was low [mean difference (MD) = −0.35%]. In the subgroup analysis, although CC did not differ from other DM diets, the association was maintained when it was compared to dietary education in diabetes (MD = −0.68%) (43). This strategy was similar to that of the present study, in which patients on fixed doses of insulin were educated about healthy eating in DM but did not receive strict eating plans.

Since CC makes feeding more flexible (18,23,24), its impact on weight has also been studied (29), and it could be inferred that it leads to increased weight. However, most RCTs have not found a change in weight or BMI after CC, and no difference in weight variation compared to controls was found at the end of these studies (16-18,20,26,42). BMI was reduced in the CC group and increased in the control group in only one RCT, with a modest but significant difference between the groups (23). Although the authors could not provide a concrete explanation for this finding, they suggested that CC might provide weight loss benefits, such as improved nutrition or increased physical activity. These data partially corroborate those of the present study, since we also observed less weight variation in the CC group despite our observational design. This difference (≈0.4 kg), although statistically significant, is not expected to have a clinical impact. However, the fact that CC resulted in better glycemic control without greater weight variation than the use of fixed doses of insulin is a relevant result, since body weight has an impact on the cardiometabolic profile (25).

The literature on patient adherence to CC lacks uniformity regarding assessment, a gold standard method, or a method that has been validated for the Brazilian population. In a Brazilian cross-sectional retrospective study on self-reported adherence to different T1D diets, 626 of the 967 patients engaging in CC reported being adherent (44). These data corroborate our results, since we estimated adherence in 69.2% of consultations in CC.

This study has some limitations. Its retrospective design does not exclude the possibility of bias, since the measurements were performed during routine consultations. Although adjustments were made, the observational design may have led to a confounding bias. The fact that we did not use suitable adherence questionnaires also limits our data on this topic.

However, our study also had a number of strengths. The sample selection was not biased, since all eligible patients with nutritional consultations between 2014 and 2018 were included. Given that all patients were treated by the same dietitian, we believe there was good uniformity of care. Although the study was observational, the favorable effects of CC were verified during a longer follow-up period (median ∼2 years) than most RCTs [duration between 3.5-30 months; only two > 1 year (17, 20)].

We can conclude that, as a nutritional strategy, CC had a positive impact on the glycemic control of patients with T1D treated in the Brazilian public health system, resulting in less body weight variation than conventional nutritional monitoring. We also found that greater effort should be made so that more patients can benefit from this technique.
